# Experiences in receiving financial incentives to access HIV care in Johannesburg, South Africa

**DOI:** 10.4102/sajhivmed.v23i1.1426

**Published:** 2022-11-17

**Authors:** Sara Rachel Schlehr, Leanne Singh, Athini Nyatela, Sizwe Nqakala, Samanta T. Lalla-Edward

**Affiliations:** 1 Department of Interdisciplinary Social Sciences, Faculty of Social and Behavioral Sciences, Utrecht University, Utrecht, the Netherlands; 2 Department of Research Development, Ezintsha Research Centre, Johannesburg, South Africa

**Keywords:** HIV care continuum, financial incentivisation, qualitative cross-sectional study, people living with HIV, behavioural nudge

## Abstract

**Background:**

Financial incentivisation has been used to improve all steps of the HIV cascade with varying results. Most studies conducted on the matter are of a quantitative nature, not giving enough space for in-depth understanding as to why financial incentives work or do not work.

**Objectives:**

To describe experiences with, and opinions on, the use of financial incentives to promote linkage to and retention in care from the perspective of people living with HIV.

**Method:**

We performed a qualitative cross-sectional study. In-depth interviews were conducted with adult men and women with HIV accessing health services or research study visits. After codebook development, NVivo 12 software was used to code and analyse the data.

**Results:**

Through the provision of financial incentives, participants were able to cover basic needs. However, some deemed financial incentives as a form of income rather than a nudge to spark interest in changing their health behaviour. Participants communicated that a need for some type of incentive exists and recommended food vouchers as the best possible solution.

**Conclusion:**

Financial incentivisation can facilitate engagement in the HIV care continuum through providing support to people living with HIV.

**What this study adds:**

This study complements the body of research that explores the feasibility of using incentives and which of them may be most beneficial in encouraging patients with HIV to enter into and sustain HIV care.

## Introduction

There are 37.7 million HIV-positive individuals globally, 7.8 million of whom are from South Africa.^1,2^ Prompt linkage to care (LTC) and initiation on antiretroviral treatment (ART) are necessary to facilitate viral suppression, which is a key strategy to control the HIV epidemic.^3,4,5,6^ Since the Joint United Nations Programme on HIV/AIDS (UNAIDS) and WHO introduced their 90-90-90 targets (90% of all HIV-positive people know their status, 90% of those who know are on ART, 90% of those who are on ART are virally suppressed), there has been significant improvement in achieving these targets in eastern and southern Africa but viral suppression showed the least improvement.^7,8^ To boost the uptake of the HIV cascade, different strategies have been employed.^9,10,11^ One systematic review identified financial incentivisation as a promising tool.^12^ Iguna et al. highlight that most research into the effects of financial incentives is quantitative, without considering the recipients’ experience.^13^ The lack of sufficient qualitative evidence that considers experiences of patients receiving financial incentives should be addressed to understand why financial incentivisation works in some environments.

In South Africa 85% of people living with HIV (PLHIV) know their status, 71% of whom are on ART.^14^ This suggests there is a gap between testing and LTC, which needs to be closed to rapidly initiate PLHIV on ART, as advised by the universal ‘test and treat’ policy.^15,16^ PLHIV who are lost to follow-up have poor outcomes.^17,18^ PLHIV need to be linked to care promptly to initiate ART, as those who are lost in these stages tend to present late.^19^ Failing to swiftly enter into ART poses a threat to the successful end of the HIV epidemic.^20^

Incentivisation has been used to encourage patients to adopt a more accountable attitude towards their health. Pettifor et al. mention two main types of incentive programmes: (1) incentives targeting upstream factors (social, economic and macrolevel factors that affect health), like poverty, and (2) incentive programmes exchanging cash for desired behaviour.^21^ Interventions targeting upstream factors, which are part of the social determinants of health,^22^ have been shown to be more successful.^21,23^ Given South Africa’s high unemployment rate of almost 35.0%^24^ and food insecurity rate of 38.5%,^25,26^ targeting these upstream factors may be a feasible way to boost engagement in the HIV cascade.

Financial incentivisation has become a popular strategy in different fields of HIV care and in other diseases with varied results.^27^ Yotebieng et al. conducted a study on prevention of mother-to-child HIV transmission (PMTCT) and concluded that small financial incentives improved the uptake of available services and boosted retention in the PMTCT cascade.^28^ El-Sadr et al. found a link between financial incentivisation and viral suppression in PLHIV.^29^ However, a Ugandan study found no effect of financial incentives on viral suppression,^30^ as did a study on improved linkage and retention of newly diagnosed PLHIV in Cape Town, South Africa.^31^

Such conflicting results cannot be fully understood solely by an analysis of quantitative data. Understanding why, in some environments, financial incentivisation improved the engagement in the HIV care continuum, and why it failed in others, may be hidden in patients’ own opinions, feelings and experiences. To comprehensively understand PLHIV’s views on financial incentivisation, we conducted a cross-sectional qualitative study of individuals who participated in a quantitative study on the effects of financial incentives and sustained linkage to HIV care (LinkMe2Care study, HSTAR013).^32^ Our aim is to present their motivations to link to and remain in care, as well as their opinions on and experiences with financial incentives.

## Methods

This study set out to learn about participants’ perspectives, opinions, and recommendations on the use of financial incentivisation to achieve linkage to and retention in care.

### Design

The study was a qualitative cross-sectional study of adult male and female PLHIV accessing health services and attending research study visits.

### Setting

This study was conducted at sites selected by the Ezintsha Research Centre for the LinkMe2Care study in the inner city of Johannesburg, South Africa, and adjoining areas in Alexandra, Soweto and Yeoville.

The inner city of Johannesburg is a unique environment with high prevalence of tuberculosis and HIV. With its dense population of refugees and economic migrants, the inner city of Johannesburg is synonymous with sex work, drugs, and crime. However, the area has many healthcare facilities and is marked with extraordinary progress towards rehabilitating disadvantaged districts.^33^

### LinkMe2Care study overview

**FIGURE 1:**
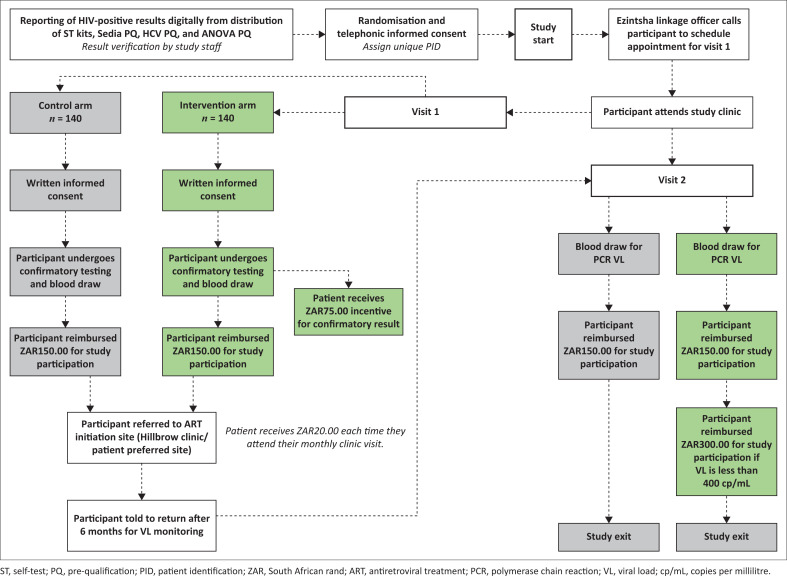
Study design workflow.

Figure 1^34^ shows the LinkMe2Care study flow.

### Recruitment and study population

Participants from LinkMe2Care were recruited from existing research studies and they were grouped according to the study channel from which they originated: Self Testing Africa HIV Self Screening (STAR HIVSS), Anova, the Hepatitis C and Sedia usability and performance studies. Recruitment for LinkMe2Care was meant to only be from STAR HIVSS however, since the study was being conducted during the coronavirus disease 2019 (COVID-19) lockdown, there were recruitment challenges posed by the restrictions that were placed on the movement and gathering of people. Therefore, recruitment was extended to include eligible participants from other studies also being conducted in the research setting at that time. From those participating in LinkMe2Care, convenience instead of systematic sampling was used to identify potential interview participants for this qualitative study. As people completed the LinkMe2Care study they were invited to participate in our qualitative interviews.

All suitable individuals were telephoned by the research team and invited to participate in the interviews. Younger men and women were prioritised in line with the HIVSS priority groups.

### Data collection

Data were collected by two trained interviewers who were fluent in several of the South African official languages. Semi-structured interview guides were used. The interview guides were piloted for six interviews which were all included in the analysis.

The exit interviews were conducted between July and August 2021 and each interview lasted 18–35 min. Given the circumstances of the COVID-19 lockdown, all interviews were conducted via mobile phone. Recording devices were used to record the audio from the phone calls. The recordings were then transcribed by an independent transcriber and, where needed, translated into English. The research team was responsible for the quality of all transcripts.

Demographic information was extracted from the LinkMe2Care database and where data were not available, participants were contacted to collect the missing information. In cases where participants could not be reached, a ‘no response’ was recorded.

### Data analysis

As data were collected, transcribed, and reviewed, five researchers independently coded four transcripts to develop the initial codebook. The initial codebook was then used by a researcher to guide the coding of the interviews and was expanded upon by including new codes where needed. Once the final codebook was created, coding was completed by two independent coders and the thematic analysis from Braun and Clarke^35,36^ was employed to search for commonly communicated feelings, opinions or experiences on different topics covered in the interviews. Thereafter, these were reported on as the key themes. NVivo 12 software was used.

### Ethical considerations

Ethical approval was received from the Human Research Ethics Committee of the University of the Witwatersrand (ethics reference: 191121) and the Research Committee of Johannesburg Health District (District Research Committee [DRC] reference: 2020-09-007). All participants provided consent for interview participation and audio recording.

All of the participants signed consent forms. Participants received R150.00 as reimbursement for their time. The interviews were conducted telephonically, and therefore all of the data are stored solely electronically on a password-restricted server in password-protected folders.

## Results

### Participant Characteristics

Altogether, 26 interviews (6 pilot and 20 study) in the participants’ language of choice were conducted. Participant characteristics are shown in Table 1. An equal number (*n* = 13) of participants from the intervention and control arms of LinkMe2Care participated in the exit interviews. The majority of participants entered from the Sedia study. Most of the participants were in the 25 to 44 years age range. There were fairly equal numbers of male and female participants. Most of the participants were unemployed.

**TABLE 1:** Characteristics of the 26 enrolled participants.

Category	Participant characteristics (*N* = 26)	
	Number	Percentage (%)
**Study channel**		
STAR HIVSS	6	23.1
Sedia	17	65.3
Hepatitis C	2	7.7
Anova	1	3.8
**LinkMe2Care arm**		
Intervention (incentivised at each study step)	13	50.0
Control	13	50.0
**Age**		
25–34	9	34.6
35–44	10	3.5
45–54	5	19.2
55+	2	7.7
**Gender**		
Female	12	46.1
Male	14	53.8
**Education**		
Less than high school	1	3.8
Some high school	12	46.1
High school graduate	4	16.4
Some college or specialised training	1	3.8
College or university graduate	4	16.4
No response	4	16.4
**Employment status**		
Unemployed	15	57.7
Employed	7	27.0
No response	4	16.4

Note: The 26 participants described in the study channel rows were all enrolled into the LinkMe2Care study.

STAR HIVSS, Self Testing Africa HIV Self Screening.

### Key themes

The majority of the participants in the control arm of LinkMe2Care misinterpreted their study reimbursement as incentives whereas participants who received the intervention understood this to be amounts additional to the study participation reimbursement. Based on this interpretation, in the majority of instances we did not see differences between the two groups in their perceptions of incentives.

Four key themes emerged: (1) perception of financial incentivisation, (2) financial incentivisation as a facilitator of the HIV care continuum, (3) financial incentivisation as a supplement to income and (4) recommendations on how to improve incentivisation-based programmes. All these themes are presented and discussed.

#### Perception of financial incentivisation

This theme demonstrates participants’ opinions on financial incentives being used as a tool to improve the individual’s engagement in the HIV care continuum. Three main perceptions arose from the analysis.

First, many participants deemed financial incentivisation as a necessity to motivate them to join or continue with the study. Participants noted that the financial incentives allowed them to pay transport and food costs that they would incur once they entered the study. Participants explained that financial reimbursements would allow them to afford better nutrition:

‘It is necessary for instances like you had called someone to come and you find that person you called wants to come but they do not have money for transport you see *mara*? … This is where it helps okay it is not that much but at least when I pass town, I am able to get something to eat like meat you see?’ (Male, 33 years old, Control)

‘Yes, the way I see it, it is important that people must be given that money because a person is able to get transport and be able to be motivated to attend the study when they are needed there’. (Male, 55 years old, Control)

‘Yes, it is to other people because some people are staying far away from places like Sebokeng in the Vaal area. They need it to be able to come to you guys, they also need the money to eat’. (Male, 30 years old, Control)

Second, at least half of the participants across both groups disclosed that they perceive financial incentivisation as a reward on their journey to better health. Although the financial incentives were appreciated by those that received them, this was not seen as a primary incentive. These participants were more motivated by their desire for improved health outcomes. One person went so far as to suggest that incentives should be used to attract people into care and then removed once they understand that they are responsible for their own care:

‘Like, I think it would depend on an individual because we all think the same. On my side, I would continue because this is for my health, I am not doing it for money. Yes, when the money is there, that is a bonus, I take it as a bonus’. (Female, 40 years old, Intervention)

‘No, it didn’t have anything to do with joining the study, it was just a bonus’. (Female, 31 years old, Intervention)

‘In most cases, it’s to encourage. I think it is. It makes a person eager to start. Along the process, you wean them off and make them aware that they don’t need to do this for the money, this is my life, I am being accountable for my responsibilities’. (Female, 31 years old, Control)

Third, a little more than a third of the participants considered financial incentives as being ineffective in linking patients to and retaining them in care. For these participants, financial incentives were seen as a negative motivator. In their opinion, people should be determined to join a study such as this with the intent of regaining their health, and taking responsibility for themselves:

‘Yeah people should not go to studies chasing money, they must join based on their health, because they want to take care of their health. What I am trying to say is that money mustn’t motivate them to join studies’. (Male, 49 years old, Intervention)

‘What is important *sisi* is that each individual must take care of themselves. Sometimes, these incentives [*programmes*] do not work, what remains important is people taking an individual initiative to take care of themselves. … So, that is why I am saying money doesn’t always work because people pay attention to the money, whereas they need to pay attention to their health’. (Male, 39 years old, Intervention)

#### Financial incentivisation as a facilitator of the HIV care continuum

People living with HIV in South Africa experience a range of different barriers to healthcare^31^ which can result in both intentional and unintentional neglect of their health. The main barriers to HIV healthcare are the costs of transportation to and from the clinic, healthcare costs and low income.^32,33^ This theme uncovers the perceived benefits of financial incentivising captured in the interviews.

Participants often mentioned that without the financial incentives they would not be able to join the study due to the lack of financial means necessary to remain in the study. By receiving monetary reimbursements, it was no longer difficult to secure basic needs for food or transport to and from the healthcare facility, all of which are vital to successfully adhere to ART. This theme illustrates how financial incentivisation facilitated opportunities which participants did not have before financial incentivisation:

‘Like I said in the beginning, when you do not work, it does help a lot because you can buy food so you can take your medication well’. (Female, 40 years old, Intervention)

‘It is helpful for transport like me. I am from Thokoza. I take three taxis to come here, so it is necessary’. (Male, 34 years old, Control)

‘It is a good thing for those who are not employed so that this money can help them buy some things so that they can be able to boost their immune systems like buying food and for transport things like that. Yes, it should be done’. (Female, 40 years old, Control)

One participant noted the value in remaining adherent to being able to getting a better incentive:

‘The time I came back for my six months I came with my sister she got R150 and got again R300 for checking her blood for viral load then they saw that her viral load is okay then she got R300 on top of the R150 she received and that thing also motivate and I think for me it was because my viral load was not okay because I was not taking my treatment correctly so I saw that next time as I am trying to push and do the right thing so that next time when they call me they must find my viral load low so that I get something’. (Female, 28 years old, Control)

#### Financial incentivisation as a supplement to income

Financial incentivisation, apart from its potential to mitigate some barriers to engaging in HIV care, became a source of income to some participants. At times participants were quite honest in acknowledging that the opportunity to earn money was their sole motivation for joining the study. Moreover, participants elaborated that without financial incentives earned from study participation, they would not be able to afford their everyday needs:

‘The fact that I knew that when I am done, I will get money. Because I am unemployed, I know I will be able to get myself some vegetables and food with the money, that’s why I was motivated. I know that I will ask someone to lend me some money for transport because I catch two taxis. I live in Soweto, but I know that I will get money and I will have leftover for vegetables’. (Female, 55 years old, Intervention)

‘You continue collecting your treatment because you know that there is a study that is going to give you money for that should you go’. (Female, 42 years old, Intervention)

‘Yes, it is necessary because it is a motivation and frankly speaking people would not come if it were not for the money including me. I would have not joined the study if there was no money. I am not going to lie’. (Male, 34 years old, Control)

#### Recommendations on how to improve incentivisation-based programmes

Many participants suggested food parcels and vouchers as improvements to the incentivisation scheme. Food parcels were recommended as a means to providing basic foods that could be made available to participants without them having to make a further trip to the store to purchase them:

‘Perhaps food parcels. I am thinking about food parcels because that is something everyone can get. Just small things like vegetables, sugar, maize meal, rice, just basic things’. (Female, 40 years old, Intervention)

‘What I think you can improve on is that you can give us food vouchers so that if I find out that I am positive I can get a voucher to have food that will last me the whole month’. (Male, 33 years old, Control)

Furthermore, food parcels and vouchers increased the likelihood that these incentives would positively influence engagement in the HIV cascade. The need to eat before taking the medication was the predominant concern, because taking ART before meals can cause discomfort.

‘People could get food parcels, any kind. This will enable them to eat before taking the medication, unlike sometimes when there is nothing to eat and you have to take your treatment. You ran out of food. And one must take their medication’. (Female, 55 years old, Intervention)

‘It would be better to provide food because you can’t take your treatment while hungry. That is what I think would be motivational’. (Male, 43 years old, Intervention)

‘You come back with positive results, you need to take medication, you can’t take medication on an empty stomach. You need a little bit of something to sustain the medication in your stomach. So, vouchers that someone can go buy something where there won’t be change back; if you get a R150 voucher, you need to take things that are limited to that R150’. (Female, 31 years old, Intervention)

However, a minority of participants did not recommend any other solutions as they thought that financial incentives are appropriate. Some reasons offered for these opinions were that money would reduce dishonest practices like selling food that was given to them for a profit, and ensure study participation. Others cited simply having faith in the success of the existing behavioural nudge programme and felt that there was no need to change it:

‘No. Money [*laughs*]. I’ll give you my honest opinion, you said there’s no right or wrong answer, so keeping it honest. Since I was going through this period, I call it surviving by the grace of God, you go for help somewhere, some organisations give out clothes, some will give food parcels. What happened is that people would take those food parcels and then they would sell them, so that contradicts. I mean they tell us that they can’t give us money on hand, but they’re going to sell that food that you gave them to someone who has money. I still think that money is a better solution because if you don’t pay then they won’t show up’. (Female, 34 years old, Intervention)

‘No, I don’t think I can recommend anything else. I think you guys are doing a great job. I think I told the other guy that I don’t think I have any recommendations because I believe that you are doing a good job’. (Male, 44 years old, Intervention)

## Discussion

This study aimed at understanding and evaluating participants’ experiences of opinions about and feelings towards financial incentivisation to improve linkage to and retention in care. To obtain this, we conducted exit interviews with adult PLHIV after they completed the LinkMe2Care study. Participants shared their motivations, opinions, and experiences with financial incentives. Besides perceptions and opinions directly related to the value of financial incentives in healthcare, and in particular HIV care, other themes also emerged. We discovered that while financial incentives were mostly well received by the participants, it was not the only reason they chose to enrol in the LinkMe2Care study.

It was evident that participants viewed financial incentives as a means to fulfil the needs required for them to link to care and maintain their ART routines. Participants in our study were clear about the fact that receiving financial incentives helped them to pay for their transport to and from the clinics, as well as the food required to meet their nutritional needs. Ghose et al. found a similar trend, and explain how patients repeatedly expressed being in favour of financial incentives as it helped them to pay for their food over and above maintaining them in HIV care.^37^ Participants expressed an awareness of how financial incentives could assist them to fulfil their unmet needs. By contrast another South African study showed that both patients and providers generally did not link financial incentivisation with structural barriers to the HIV cascade like transport costs or food insecurity, but rather with individual factors like intrinsic motivation.^38^

Even though most participants thought financial incentives were a positive influence on linkage and retention, a few participants expressed that financial incentivisation should not be used in the HIV care continuum because being responsible for one’s health behaviour is the most important factor. This finding is supported by Clouse et al.’s findings from a study on acceptability and feasibility of financial incentives among pregnant South African women, where it was found that participants deemed health education and counselling to be more important than financial incentives.^39^ Health accountability is an important factor in the HIV care continuum as was similarly shown in a Ukrainian study on patients and provider perspectives on improving LTC.^40^ Robbins and Dunn emphasise that responsibility drives and sustains change.^41^ A study from Kenya reported that participants who were intrinsically motivated to engage in care were less prompted by financial incentives.^13^ An important area for future research would be to consider interventions that target replacing patients’ dependence on financial incentives with non-incentivised self-management of their health. For instance, the benefits of combining education and self-management were demonstrated in the systematic review of the teach-back method applied on people with chronic diseases. This study showed improvement in adherence, self-care, and disease-specific knowledge.^42^ Given that over a third of participants place their health above financial incentives and view financial incentives as no more than ‘a bonus’, it would be beneficial to investigate how a similar attitude can be instilled in PLHIV who neglect their health.

Some PLHIV see financial incentives as a gateway to an improved lifestyle not necessarily linked to the desire for health improvement or to the study. For these participants, financial incentives are a way to earn an income or supplement an existing one. Based on the high unemployment rate in South Africa,^24^ we think the main driver of this behaviour lies within this particular upstream factor combined with a relatively low standard of living compared to other country members of the Organisation for Economic Co-operation and Development.^21,43^ Therefore, we suggest new interventions focusing on upstream structural factors like unemployment or low education standards to improve engagement in the HIV care continuum.

Drawing on the participants’ motivations, this study showed that a demand for some type of incentive or support is still in place. Participants expressed a need for an improvement or adjustment in the incentivisation programmes. For them this can be achieved by promoting non-financial incentives, like food vouchers. According to them, this may alleviate the possible physical stress to the body that is caused by not eating properly before taking HIV treatment.

However, McCoy et al., in their Tanzanian study, point out that distributing food baskets as a form of incentive may be harder than distributing cash, and therefore less effective.^44^ This is a reminder that while other forms of support beyond money and food can be effective, they may not necessarily be able to be applied in all contexts. Kennedy et al.’s systematic review on the uptake of medical male circumcision for HIV prevention, particularly as it pertains to awarding food or transport vouchers, exemplifies this.^44^ To avoid distribution problems, we suggest that vouchers for specific foods or amounts that limit spend to particular items, within an accessible South African grocery store, should be distributed instead of direct cash transfers.

### Strengths and limitations

Despite completing the study during COVID-19 lockdown, we were still able to conduct the research with a sufficient number of participants to reach data saturation. The semi-structured interviews allowed the researchers to ask additional questions uncovering more in-depth information regarding financial incentives.

COVID-19 restrictions (particularly around movement and social distancing) hindered the ability to access and conduct in-field recruitment. This together with the failure to achieve high self-reporting among STAR HIVSS participants negatively impacted the implementation of the LinkMe2Care study, which prompted the team to include more recruitment channels as reported. Due to the necessitated deviation from the original protocol of the LinkMe2Care study, participants were also recruited from different study channels (Sedia and Hepatitis C usability and performance studies, ANOVA) and had varying knowledge about financial incentivisation. The STAR HIVSS recruits had a generally better understanding of the study compared to others. However, only six STAR HIVSS recruits participated in the exit interviews compared to 17 Sedia recruits which was not desirable due to the differences in knowledge about financial incentivisation. To add to that, some participants were part of multiple studies, confusing which study they were being contacted about, and ultimately resulting in mixed messages. However, when this was noticed early on in the recruitment for the exit interviews and data collection processes, as mitigation, the research team developed a second interview guide for particpants who did not enter LinkMe2Care through STAR HIVSS so that the questions asked would still yield some of the information we were seeking to find.

### Recommendations

To boost the engagement in the HIV care continuum, we recommend that public health professionals and policymakers consider interventions that target upstream factors in addition to offering financial incentives to catalyse responsible health-related behaviour adjustments. We also suggest that researchers conduct studies into the effects of other types of incentives, namely food or travel vouchers, based on the findings of this study.

## Conclusion

Three specific opinion sub-groups emerged: (1) participants who deemed financial incentives as unimportant and regarded them as just ‘a bonus’ in their journey to better health, anticipating they did not need the nudge to enter into the HIV cascade, (2) participants who considered financial incentives as imperative as they provided for their unmet needs and created an opportunity to start caring about their health, and (3) participants who joined the study solely on the premise of cash incentives without taking into consideration their health. Based on the distribution of the opinions we conclude that financial incentives can facilitate engagement in the HIV care continuum through providing support to PLHIV. This support facilitates participants meeting basic needs like food and transport costs which are necessary to link patients to care and sustain them in ART.
